# Occupational Health: Analysis of the Psychological Impact of Exposure to Needlestick and Sharps Injuries Among Primary Health Care Professionals

**DOI:** 10.1155/nrp/4002291

**Published:** 2026-07-29

**Authors:** Talita Priscilla da Silva Guerra, Rodrigo Pierobon Rodrigues, Roberto Martins de Oliveira, Fernanda Lopez Rosell, Caio Vieira de Barros Arato, Luís Eduardo Genaro

**Affiliations:** ^1^ Anhanguera College of Jundiaí, Jundiaí, São Paulo, Brazil; ^2^ Faculdade de Odontologia de Piracicaba, Universidade Estadual de Campinas (Unicamp), Piracicaba, São Paulo, Brazil, unicamp.br; ^3^ São Paulo State University (UNESP), School of Dentistry, Araraquara, São Paulo, Brazil, unesp.br

**Keywords:** health professionals, occupational accidents, occupational health

## Abstract

Occupational exposure to biological material, particularly needlestick and sharps injuries, is a relevant public health concern in Primary Health Care (PHC), as it combines infectious risk with psychological sequelae that are often underestimated. This study aimed to analyze the occurrence of needlestick and sharps injuries and their psychological impact among PHC professionals, considering biosafety knowledge, vaccination status, postexposure conduct, and perceived availability of psychological support. This was a descriptive, exploratory, mixed‐methods study conducted in a medium‐sized municipality in the interior of São Paulo state, Brazil. A nonprobabilistic convenience sample of 53 PHC workers was included, comprising nursing technicians, nurses, oral health assistants, and dentists. Data were collected using a sociodemographic questionnaire, a biosafety and postexposure management instrument, the Impact of Event Scale–Revised (IES‐R) among injured participants, and open‐ended questions analyzed using the Collective Subject Discourse technique. Among the 53 participants with valid responses for injury occurrence, 16 reported having experienced a needlestick or sharps injury, corresponding to 31.4% of the valid sample. Dentists presented the highest proportion of reported injuries, followed by nursing technicians, oral health assistants, and nurses. Self‐reported hepatitis B vaccination coverage was high, with 94.3% reporting completion of the three‐dose schedule; however, insufficient anti‐HBs testing and limited guidance regarding postvaccination serological monitoring indicated weaknesses in occupational health surveillance. Exploratory bivariable and multivariable analyses suggested higher odds of injury among dentists and among professionals who reported feeling vulnerable to acquiring diseases although these associations did not reach statistical significance and presented wide confidence intervals. Qualitative findings indicated that needlestick and sharps injuries were frequently associated with fear, anxiety, insecurity, sleep disturbance, increased vigilance, and changes in safety practices. Although participants recognized the importance of psychological support after occupational exposure, formal psychological follow‐up was rarely reported. Among injured professionals, the IES‐R total score indicated relevant psychological distress. Using the ≥ 33 cutoff, 37.5% presented clinically significant post‐traumatic stress symptoms, while 43.8% reached the ≥ 24 threshold for elevated psychological distress. No statistically significant predictors of IES‐R scores were identified; however, these findings should be interpreted cautiously due to the small number of injured participants and limited statistical power. In conclusion, needlestick and sharps injuries in PHC should be understood not only as biological exposure events but also as potentially traumatic occupational experiences. As a local exploratory study with a nonprobabilistic sample, the findings should not be generalized without caution. Nevertheless, they highlight the need for structured institutional actions, including periodic biosafety training, clear definition of occupational exposure, systematic reporting, immediate referral to reference services, anti‐HBs monitoring, postexposure serological follow‐up, nonpunitive reporting systems, and incorporation of psychological screening or referral into postexposure care pathways.

## 1. Introduction

Occupational accidents involving exposure to biological materials constitute a public health problem, with consequences that affect both health workers and the institutions in which they work [1, 2]. These events are characterized by direct contact with body fluids and may occur through percutaneous inoculation by sharps or needlestick injuries, as well as through contact with mucous membranes or nonintact skin [3, 4]. Globally, needlestick and sharps injuries remain among the most relevant occupational hazards affecting healthcare workers. Recent literature reports that more than 2 million occupational exposures occur annually among approximately 35 million healthcare workers worldwide, reinforcing the global magnitude of this problem and the need for systematic prevention strategies [5]. In the United States alone, the Centers for Disease Control and Prevention estimates that approximately 385,000 needlestick and other sharps‐related injuries occur annually among hospital‐based healthcare personnel, with similar events also occurring in other healthcare settings, such as clinics, long‐term care facilities, emergency services, and home care [6].

From an occupational health perspective, these injuries are particularly relevant because they are not isolated technical events but indicators of broader working conditions, including safety culture, training, workload, availability of protective devices, and institutional surveillance [6–10]. The World Health Organization emphasizes that health workers are exposed to multiple occupational risks, including infections, injuries, psychosocial hazards, violence, and inadequate working conditions; therefore, worker protection should be considered a core responsibility of health systems [11].

The pathogens associated with these exposures include human immunodeficiency virus (HIV) and hepatitis B and C viruses. Their prevalence among source patients in health services may vary, with Brazilian studies reporting reactive serology in roughly 1%–6%, depending on the care setting [12, 13]. However, the consequences extend beyond biological risk, as such events can trigger relevant psychosocial impacts, affecting the mental and emotional well‐being of health workers and often leading to disorders such as anxiety, sleep disturbances, and even postexposure suicide [14–18]. This psychological burden may be intensified during the waiting period for serological results, when workers face diagnostic uncertainty, fear of infection, concerns about stigma, and possible repercussions for family and social relationships. Thus, needlestick and sharps injuries should be understood not only as biological exposures but also as potentially traumatic occupational events [16, 19–23].

From a theoretical perspective, needlestick and sharps injuries may be understood as potentially traumatic occupational events because they combine sudden exposure, perceived threat to health, diagnostic uncertainty, fear of infection, and loss of perceived control over one’s own safety. These elements are consistent with psychological models of traumatic stress, in which the subjective appraisal of threat and the persistence of uncertainty may contribute to intrusive thoughts, avoidance behaviors, hyperarousal, sleep disturbances, and emotional distress [16, 19–21, 23]. In PHC settings, these reactions may be intensified by decentralized care, limited immediate support, uncertainty about postexposure pathways, and insufficient institutional recognition of psychological suffering after exposure [22, 24–26].

The mental health dimension of occupational exposure is increasingly relevant in the global debate on worker safety. The WHO guidelines on mental health at work emphasize that psychosocial risks, including heavy workloads, unsafe working environments, low job control, and insufficient organizational support, can negatively affect workers’ mental health [11, 27]. In addition, the WHO and ILO have emphasized that depression and anxiety are responsible for approximately 12 billion lost working days each year, with major social and economic consequences [28, 29]. In this context, the psychological sequelae of needlestick and sharps injuries require greater attention, particularly in health systems where postexposure protocols focus mainly on biological prophylaxis and administrative reporting, while emotional follow‐up remains poorly structured [19, 21, 23].

Beyond immediate distress, psychological sequelae after occupational exposure may affect professional confidence, perceived safety, work engagement, absenteeism, burnout, and workforce retention. Previous evidence indicates that occupational exposure to biological material and sharps‐related injuries may affect workers’ psychological well‐being, quality of life, perceived safety, and professional functioning. Therefore, the impact of needlestick and sharps injuries should be considered not only as an individual mental health outcome but also as an occupational health and workforce sustainability issue [11, 19, 21–23, 27–31].

Multiple factors contribute to the occurrence of these accidents, including inadequate or absent use of personal protective equipment (PPE), excessive workloads, staffing shortages, insufficient training, and inappropriate clinical practices [32, 33]. These factors not only increase biological risk but also impose considerable psychological stress, affecting professionals’ quality of life and productivity and interfering with their personal and social lives [30, 31]. Moreover, when occupational exposures become normalized in daily practice, workers may underestimate certain events, such as splashes of body fluids, and may fail to notify them or seek appropriate postexposure care. This normalization of risk reflects not only individual perception but also possible weaknesses in organizational safety culture and continuing education [6, 34–37].

In the context of Primary Health Care (PHC), these accidents are particularly common given the nature of procedures performed and the range of professionals involved, including physicians, nurses, and dentists [38–40]. PHC professionals may be exposed to blood and body fluids during vaccination, wound care, dental procedures, home visits, emergency care, medication administration, and handling of contaminated materials. Evidence from primary healthcare settings has shown that occupational exposure to blood and body fluids remains frequent and may be substantially underreported, reinforcing the need for structured reporting and postexposure management systems [41]. Unlike hospital settings, PHC services may present specific challenges, such as decentralized care, variable availability of safety‐engineered devices, limited access to immediate referral services, and insufficient institutional follow‐up after exposure [6, 42]. In dental practice, exposure to blood and blood‐contaminated saliva is also a relevant occupational risk, and gaps in reporting practices have been described among dental professionals [43, 44].

These professionals report high levels of anxiety, distress, and fear, especially during the immunological window period while test results are pending. Uncertainty about outcomes, social stigma, and lack of institutional support may further intensify psychological suffering [20, 26]. Despite attention to pharmacological prophylaxis and administrative reporting of these accidents, many occupational health services fail to provide ongoing psychosocial support or emotional follow‐up protocols [45]. Therefore, strengthening the literature on this topic is essential because the burden of needlestick and sharps injuries cannot be fully captured by infection risk alone; it must also include psychological distress, risk perception, postexposure behavior, and institutional support [6, 16, 19–21, 46, 47].

Therefore, this study aimed to analyze the psychological sequelae and impacts of needlestick and sharps injuries among PHC professionals, as well as their level of knowledge regarding biosafety and occupational exposure, and the availability and perceived adequacy of psychological support.

## 2. Methodology

### 2.1. Ethical Considerations

The study was approved by the Human Research Ethics Committee of Anhanguera (CAAE: 86216125.5.0000.0347). The reporting of this observational study was guided by the Strengthening the Reporting of Observational Studies in Epidemiology (STROBE) statement [48].

### 2.2. Study Setting

The research was conducted in a medium‐sized municipality in the interior of the state of São Paulo, Brazil, located in the Campinas Metropolitan Region, approximately 80 km northwest of the state capital.

### 2.3. Sample

The sample comprised health professionals from four distinct categories working in the municipality’s PHC units. These categories were selected due to their professional roles and occupational exposure to needlestick and sharps injuries in the services. A nonprobabilistic convenience sample was used, including professionals available during the data collection period. Participants were dentists (*n* = 7), oral health assistants (*n* = 11), nursing technicians (*n* = 20), and nurses (*n* = 15). Participants were contacted by telephone to schedule the date and time for questionnaire administration.

Given the limited number of professionals available in the municipal PHC network and the exploratory nature of the study, this research was designed as a local mixed‐methods exploratory study. Its purpose was not to produce generalizable estimates for all PHC professionals but to identify preliminary patterns of occupational exposure, psychological sequelae, perceived risk, and institutional support needs.

Inclusion criteria were health professionals directly involved in patient care in PHC and a minimum of 6 months of experience in PHC to ensure sufficient familiarity with work routines and occupational risks. Only those who voluntarily agreed to participate and signed the Informed Consent Form were included. Exclusion criteria were professionals who were on leave during the data collection period due to medical leave or other temporary reasons.

### 2.4. Pilot Study

To minimize reactivity of participants to the presence of the researcher, a pilot study was conducted with 10 participants to assess methodological adequacy, including pretesting and estimating the time required for completion. According to Lefevre et al. [49], pretesting in a pilot study is essential to verify whether the questions are fully understood. This stage also helped identify potential signs of reactivity and how it might affect responses, allowing adjustments to the approach if necessary. An environment was established to ensure participants felt comfortable expressing genuine opinions and perceptions.

### 2.5. Data Collection Instruments

Data collection included a sociodemographic questionnaire; the Biosafety, Occupational Exposure, and Postaccident Conduct Questionnaire; and the Impact of Event Scale–Revised (IES‐R), used to measure the psychological impact of needlestick and sharps injuries.

#### 2.5.1. Sociodemographic Questionnaire and Collective Subject Discourse (CSD)

Data were collected using an open‐ended questionnaire in an appropriate private room to ensure a calm environment and minimize local interference. Participants completed a self‐administered sociodemographic questionnaire, followed by an interview script containing topic‐related open questions. Confidentiality, data privacy, and anonymity were ensured. Anonymity was preserved through the use of pseudonyms, and information was used exclusively for scientific purposes. After collection, responses were transferred to a computer for transcription and subsequent analysis. Questions were formulated in an open and objective manner, as recommended by Lefevre et al. [49].

Qualitative data were analyzed descriptively and through the qualiquantitative CSD technique. According to Lefevre et al. [50], CSD is grounded in Social Representation Theory and focuses on the analysis of collected verbal material extracted from individual statements. It is based on the premise that individuals within a social group share ideas, opinions, beliefs, and expressions, which can be synthesized into a single discourse that reflects common content and arguments regarding a given issue—moving from multiple individual testimonies to a collective testimony.

The discourse was written in the first person singular to create the impression of a collective thought expressed directly by the “voice” of a single collective subject [50]. When using the software, this implies that participants’ coded identification is not displayed. This presentation is consistent with Social Representation Theory, in which collective discourse represents the externalization of social entities internalized and incorporated by individuals through everyday interactions.

The qualitative component was reported considering the Consolidated Criteria for Reporting Qualitative Research (COREQ), aiming to improve transparency regarding data collection, researcher–participant interaction, analytical procedures, and presentation of qualitative findings [51].

Because the qualitative analysis was restricted to participants who reported needlestick or sharps injuries, formal theoretical saturation was not assumed. Instead, the analysis sought to identify recurring meanings, shared perceptions, and common elements within the available injured subgroup. Therefore, the qualitative findings should be interpreted as descriptive and exploratory, reflecting the collective discourse of this specific group rather than exhaustive saturation of all possible experiences.

After data collection, each written individual statement was analyzed to capture collective thinking. Key expressions were initially identified. The guiding questions were developed by the authors based on literature addressing the topic.

##### 2.5.1.1. Guiding Questions Used During Data Collection

• Have you ever been involved in an accident with a sharp instrument?
• If yes, how did the accident happen?

If a sharps injury was reported, the following questions were asked:

• Do you have disturbing dreams that repeat the event or are clearly related to the accident? If yes, what are these dreams like?
• Do you feel distressed when something reminds you of the event? If yes, in what way?
• Did you change any safety habits after the accident? If yes, what changes?
• Did you receive any psychological follow‐up after the accident? If yes, how was it?
• What is your opinion about the need for psychological support after this type of occupational accident?
• What are your suggestions to prevent similar situations from occurring?

If no sharps injury was reported, the following questions were asked:

• Even without having experienced an accident, have you had disturbing dreams related to sharps accidents at work?
• What safety measures do you take to avoid sharps injuries?
• What is your opinion about the need for psychological support after a sharps injury?

#### 2.5.2. Biosafety, Occupational Exposure, and Postaccident Conduct Questionnaire

Data collection also included a semistructured, self‐administered questionnaire developed specifically for this study based on Lima et al. [52], Schroeder et al. [53], and Barros et al. [54]. The instrument underwent pretesting and was revised as needed to ensure clarity and applicability.

The questionnaire was organized into four thematic sections. Vaccination status was assessed based on doses received for hepatitis B, tetanus toxoid, diphtheria, measles, BCG, influenza, and oral polio vaccine (Sabin). Postvaccination hepatitis B serology (anti‐HBs) and the source of guidance to obtain it were also investigated.

Regarding biosafety, adherence to standard precautions and PPE use were assessed. Occupational exposure and workplace accidents were investigated through recognition of saliva splashes and exposure to body fluids as accidents, as well as perceived vulnerability to illness during clinical activities involving patient care. Diseases potentially acquired through cross‐infection in dentistry were assessed, including herpes simplex, tuberculosis, AIDS, hepatitis B, hepatitis C, Chagas disease, tetanus, syphilis, measles, meningococcal disease, rubella, and influenza.

Additionally, the occurrence, frequency, circumstance, severity, and type of accident were investigated, as well as the context in which it occurred. Postaccident actions were assessed, including reporting, referral to a reference service, the person responsible for referral, and reasons for nonreferral. Knowledge of postexposure protocols was evaluated as were factors influencing accident occurrence (e.g., inattention, PPE failure such as glove/mask damage, haste, pressure from supervisors, insecurity, and inadequate working conditions/physical area).

#### 2.5.3. IES‐R

The IES‐R, originally developed in English to screen symptoms and measure the impact of a specific stressful event, was applied to participants. The scale was validated for the Brazilian population by Caiuby et al. [15] and showed high internal consistency, with Cronbach’s alpha values ranging from 0.85 to 0.96, indicating reliability across different application contexts.

The IES‐R consists of 22 items rated on a Likert scale from 0 (“*not at all*”) to 4 (“*extremely*”) and includes three subscales:

• Avoidance (avoidant behaviors): Items 5, 7, 8, 11, 12, 13, 17, and 22.
• Intrusion (intrusive memories): Items 1, 2, 3, 6, 9, 16, and 20.
• Hyperarousal (anxiety symptoms and physiological activation): Items 4, 10, 14, 15, 18, 19, and 21.

The scale assessed the frequency and intensity of emotional and physiological responses associated with the potentially traumatic event, enabling a quantitative evaluation of psychological effects experienced by injured participants [15]. Subscale scores were calculated as the mean of their respective items, excluding unanswered items. The total score was computed as the sum of the three subscale scores [15, 20].

### 2.6. Data Analysis

Qualitative data were analyzed descriptively using the qualiquantitative CSD technique, with support from Qualiquantisoft®. Quantitative data were organized in Microsoft Excel and analyzed using IBM SPSS Statistics Version 20.0, adopting a 95% confidence interval (CI) and a 5% significance level (*p* < 0.05). Descriptive analyses were performed using absolute and relative frequencies for categorical variables and measures of central tendency and dispersion for numerical variables. Valid‐case analysis was adopted for inferential analyses involving needlestick and sharps injuries since two participants did not provide information on injury occurrence; therefore, analyses using injury occurrence as the outcome included 53 professionals, while analyses involving the IES‐R were restricted to the 16 injured participants. Exploratory bivariable analyses were performed using odds ratios (ORs), 95% CIs, and *p* values, with Fisher’s exact test applied when appropriate due to small cell counts.

An exploratory multivariable logistic regression model was fitted for injury occurrence, including professional category, age, and perceived vulnerability as predictors. The IES‐R was analyzed as a continuous measure and according to clinically relevant cutoff points, using ≥ 33 for clinically significant post‐traumatic stress symptoms and ≥ 24 as a sensitivity threshold for elevated psychological distress. Exploratory logistic regression was used for elevated IES‐R symptoms, and exploratory linear regression was performed using the IES‐R total score as the dependent variable. A post hoc power analysis was conducted using Cohen’s h to estimate the power for the observed difference in injury occurrence between dentists and nondentists. Due to the limited sample size, all inferential analyses were interpreted as exploratory and hypothesis‐generating.

## 3. Results

### 3.1. Sample Characteristics

A total of 53 professionals participated in the study, with a predominance of females (98.1%) and participants who self‐identified as heterosexual (96.2%). More than half reported being married (56.6%) and having children (56.6%). Regarding occupational profile, nursing technicians were the most frequent category (39.6%), followed by nurses (26.4%), oral health assistants (18.9%), and dentists (15.1%).

Length of employment in public health was mainly between 1 and 5 years (32.1%). In terms of socioeconomic profile, nearly half reported an income between 1 and 3 minimum wages (49.1%), and most self‐identified as White (77.4%). The most frequently reported religion was Catholic (37.7%), followed by Evangelical (26.4%).

To characterize the study population, sociodemographic and professional variables were summarized using absolute and relative frequencies, as shown in Table 1.

**TABLE 1:** Distribution of participants’ sociodemographic and professional characteristics (frequency and percentage).

Variable	Category	*n* (%)
Gender	Female	52 (98.1)
	Male	1 (1.9)

Sexual orientation	Heterosexual	51 (96.2)
	Homosexual	1 (1.9)
	Prefer not to say	1 (1.9)

Marital status	Married	30 (56.6)
	Single	14 (26.4)
	Separated	6 (11.3)
	Widowed	2 (3.8)
	Single (other)	1 (1.9)

Children	Yes	30 (56.6)
	No	23 (43.4)

Profession	Nursing technician	21 (39.6)
	Nurse	14 (26.4)
	Oral health assistant	10 (18.9)
	Dentist	8 (15.1)

Time working in public health	Up to 1 year	8 (15.1)
	1–5 years	17 (32.1)
	6–10 years	10 (18.9)
	11–20 years	12 (22.6)
	> 20 years	6 (11.3)

### 3.2. Vaccination Status and Gaps in Guidance

Self‐reported hepatitis B vaccination coverage was high, with 94.3% reporting completion of the three‐dose schedule. Nevertheless, 20.8% stated they had not undergone anti‐HBs testing to verify serologic response. Among those who had serology performed, most reported having it done only once after vaccination (40.5%), followed by more than once (35.7%) and every 6 months (23.8%).

Fewer than half of participants (39.6%) reported receiving guidance to perform anti‐HBs testing, mainly from their academic training (38.1%) and the workplace (23.8%). Among those who did not undergo testing, informational barriers predominated, including “did not know where to have it done” (45.5%) and “was unaware it was necessary” (36.4%). Other vaccines showed high self‐reported uptake, particularly influenza and MMR (98.1%) and BCG (94.3%).

These results indicate that high vaccination coverage did not necessarily translate into adequate postvaccination serological monitoring, revealing an institutional gap in guidance and follow‐up after hepatitis B immunization. Vaccination status, anti‐HBs testing, and selected immunization indicators are presented in Table 2.

**TABLE 2:** Vaccination status and anti‐HBs testing among participants and selected other vaccines (frequency and percentage).

Indicator	Category	*n* (%)
Hepatitis B (doses)	3 complete doses	50 (94.3)
	2 doses	1 (1.9)
	2 doses (schedule in progress)	1 (1.9)
	Not reported	1 (1.9)

Anti‐HBs performed	Yes	42 (79.2)
	No	11 (20.8)

Anti‐HBs frequency (*n* = 42)	Only once after vaccination	17 (40.5)
	More than once	15 (35.7)
	Every 6 months	10 (23.8)

Guidance on anti‐HBs	Yes	21 (39.6)
	No	32 (60.4)

Reason for not performing (*n* = 11)	Did not know where to do it	5 (45.5)
	Was unaware it was necessary	4 (36.4)
	No response	2 (18.1)

Influenza vaccine	Yes	52 (98.1)

MMR vaccine	Yes	52 (98.1)

BCG vaccine	Yes	50 (94.3)

The occurrence of injuries was unevenly distributed across professional categories. Dentists presented the highest proportion of reported injuries (55.6%), followed by nursing technicians (33.3%), oral health assistants (22.2%), and nurses (20.0%). The distribution of needlestick and sharps injuries according to professional category is shown in Table 3.

**TABLE 3:** Occurrence of needlestick and sharps injuries by professional category.

Profession	Valid *n*	Injury: yes	Injury: no	Missing
Oral health assistant	9	2 (22.2%)	7 (77.8%)	1
Dentist	9	5 (55.6%)	4 (44.4%)	0
Nurse	15	3 (20.0%)	12 (80.0%)	0
Nursing technician	18	6 (33.3%)	12 (66.7%)	1
Total	51	16 (31.4%)	35 (68.6%)	2

Bivariable exploratory analyses were performed to estimate the magnitude of associations with injury occurrence. Dentists had 3.52 times higher odds of reporting an injury than nondentists although the association did not reach statistical significance (OR = 3.52; 95% CI: 0.80–15.54; *p* = 0.118). Professionals who reported feeling vulnerable to acquiring diseases also had higher odds of reporting injury (OR = 4.02; 95% CI: 0.78–20.78; *p* = 0.103).

Although these associations were not statistically significant, their magnitude and wide CIs suggest clinically relevant tendencies and statistical imprecision, rather than definitive evidence of absence of association. To explore potential factors associated with injury occurrence, bivariable analyses were performed, as presented in Table 4.

**TABLE 4:** Bivariable exploratory associations with needlestick/sharps injury occurrence.

Exploratory association	Odds ratio	95% CI	*p* value
Dentist vs. nondentist	3.52	0.80–15.54	0.118
Dental team vs. other professionals	1.70	0.50–5.74	0.529
Feels vulnerable to acquiring diseases	4.02	0.78–20.78	0.103
Received guidance on anti‐HBs testing	1.63	0.49–5.39	0.547
Underwent anti‐HBs testing	1.28	0.29–5.66	1.000

A multivariable exploratory model was also fitted using injury occurrence as the outcome and including professional category, age, and perceived vulnerability. After adjustment, dentists continued to present higher odds of reporting injury (adjusted OR = 2.31; 95% CI: 0.45–11.81; *p* = 0.313), and perceived vulnerability remained associated with increased odds of injury (adjusted OR = 3.11; 95% CI: 0.56–17.23; *p* = 0.194).

These adjusted estimates should be interpreted cautiously because the small number of events limited model stability and resulted in wide CIs. An exploratory multivariable model was fitted to assess predictors of needlestick and sharps injury occurrence, as shown in Table 5.

**TABLE 5:** Multivariable exploratory model for needlestick/sharps injury occurrence.

Predictor	Adjusted OR	95% CI	*p* value
Dentist	2.31	0.45–11.81	0.313
Age	0.94	0.87–1.02	0.156
Feels vulnerable	3.11	0.56–17.23	0.194

### 3.3. Biosafety, Risk Perception, and Postexposure Conduct

Regarding risk perception, 45.3% considered splashes of body fluids on any part of the body to constitute an occupational accident, whereas 54.7% did not. Most participants reported feeling vulnerable to acquiring diseases during practical activities (66.0%). The diseases most frequently mentioned as possible through cross‐infection were influenza (88.7%), tuberculosis (83.0%), HIV/AIDS (79.2%), hepatitis B (75.5%), and hepatitis C (73.6%).

Among injured participants, most reported a single episode (75.0%), with 87.5% reporting the accident and 75.0% stating that they changed their work practices after the event. Referral to a reference service was reported by 62.5%.

These findings suggest that occupational exposure was frequently followed by changes in professional behavior, but postexposure referral and follow‐up were not universal.

Qualitative analysis revealed that needlestick and sharps injuries were frequently associated with emotional distress, especially fear, anxiety, insecurity, and uncertainty regarding possible biological contamination. Participants’ statements illustrated the immediate psychological impact of occupational exposure: “I was afraid at first after the accident” (P24); “At the time I felt insecure and became nervous about the accident” (P41); and “In my case, I went 2 days without sleeping properly…” (P16).

In addition to emotional repercussions, the reports also indicated behavioral changes after the accident, mainly related to greater caution and safer handling of sharps. Participants described becoming more attentive during procedures, avoiding needle recapping, and reinforcing the use of PPE: “I became more attentive” (P2); “I no longer recap needles” (P2, P19, P25); and “I stay more alert, always wearing gloves…” (P48).

Despite these psychological and behavioral consequences, formal psychological follow‐up after the accident was rarely reported: “I did not receive psychological follow‐up after the accident” (P12, P16, P26). Nevertheless, participants recognized the importance of this type of support, particularly because occupational accidents may generate feelings of guilt, fear, anxiety, and trauma: “I consider it important because of feelings of guilt, fear, and anxiety” (P5); and “It is very important because every accident causes trauma” (P14).

### 3.4. Psychological Impact Assessment and Clinical Interpretation of the IES‐R

To address the clinical interpretation of psychological impact, IES‐R scores were analyzed both as continuous scores and according to clinically relevant cutoff points. A cutoff of ≥ 33 was adopted to indicate clinically significant post‐traumatic stress symptoms, and a sensitivity analysis using ≥ 24 was also performed to identify participants with elevated psychological distress.

Among the 16 professionals who reported a needlestick or sharps injury, the IES‐R total score had a mean of 22.9 ± 15.6, median of 21.5, and interquartile range of 10.0–36.0. The mean subscale scores were 8.3 ± 5.6 for intrusion, 8.9 ± 6.6 for avoidance, and 5.8 ± 4.4 for hyperarousal.

Using the ≥ 33 cutoff, 6 of the 16 injured professionals (37.5%) presented clinically significant post‐traumatic stress symptoms. In the sensitivity analysis using the ≥ 24 cutoff, 7 of the 16 injured professionals (43.8%) presented elevated psychological distress. The distribution of IES‐R scores and the proportion of participants above clinically relevant cutoff points are shown in Table 6.

**TABLE 6:** IES‐R scores and clinically relevant cutoff points among injured participants (*n* = 16).

IES‐R measure	Mean ± SD or *n*/*N*	Median or %	IQR/interpretation
Intrusion	8.3 ± 5.6	7.0	4.8–11.0
Avoidance	8.9 ± 6.6	8.0	3.5–14.0
Hyperarousal	5.8 ± 4.4	6.0	2.0–8.8
IES‐R total	22.9 ± 15.6	21.5	10.0–36.0
IES‐R ≥ 24	7/16	43.8%	Sensitivity threshold
IES‐R ≥ 33	6/16	37.5%	Clinically significant threshold

Because some strata included only one or two injured participants, subgroup‐specific IES‐R means were interpreted only descriptively and were not considered representative of group‐level trends. Therefore, emphasis was placed on the overall distribution of IES‐R scores and on clinically relevant cutoff points among all injured participants. Mean IES‐R scores according to professional category are presented only as descriptive indicators in Table 7.

**TABLE 7:** Mean IES‐R scores by professional category among injured participants.

Profession	*n*	Intrusion	Avoidance	Hyperarousal	IES‐R total
Oral health assistant	2	8.5	16.0	5.5	30.0
Dentist	5	6.0	5.8	3.0	14.8
Nurse	3	9.7	9.0	11.7	30.3
Nursing technician	6	9.3	8.5	5.8	23.7

*Note:* Due to the small number of injured participants in each professional category, these values are descriptive only and should not be interpreted as representative group‐level estimates.

### 3.5. Predictive Analyses of Elevated IES‐R and Total IES‐R Score

Given the limited number of injured participants and the small number of events above the IES‐R cutoff, all predictive analyses were considered exploratory and hypothesis‐generating. Estimates were interpreted primarily according to their magnitude and CIs, rather than statistical significance alone.

A logistic model was fitted to explore predictors of clinically significant IES‐R scores using the ≥ 33 cutoff. No predictor reached statistical significance. The dentist category showed lower odds of elevated IES‐R scores compared with other professionals (OR = 0.24; 95% CI: 0.02–3.56; *p* = 0.298), but the estimate was imprecise due to the small number of injured participants.

A linear regression model was also fitted using the IES‐R total score as the dependent variable. The model did not identify statistically significant predictors although it explained approximately 20.3% of the variability in total IES‐R scores.

These analyses add an exploratory predictive approach to the study but should be interpreted as hypothesis‐generating because only 15 injured participants had complete data for the adjusted models. An exploratory logistic regression model was fitted to assess predictors of clinically significant IES‐R symptoms, as presented in Table 8.

**TABLE 8:** Exploratory model for clinically significant IES‐R symptoms among injured participants.

Predictor	OR for IES‐R ≥ 33	95% CI	*p* value
Dentist	0.24	0.02–3.56	0.298
Age	0.97	0.82–1.16	0.762
Years of practice	1.16	0.84–1.60	0.362

An exploratory linear regression model was also fitted using the IES‐R total score as the dependent variable, as shown in Table 9.

**TABLE 9:** Exploratory linear regression model for IES‐R total score among injured participants.

Predictor	*β* coefficient	95% CI	*p* value
Dentist	−10.18	−30.81 to 10.45	0.301
Age	0.26	−1.25 to 1.77	0.713
Years of practice	0.96	−1.46 to 3.38	0.403
Model *R* ^2^	0.203	—	—

### 3.6. Power Considerations and Interpretation of Nonsignificant Findings

A post hoc power analysis was conducted for the observed difference in injury occurrence between dentists and nondentists. Dentists had an observed injury proportion of 55.6%, whereas nondentists had an observed injury proportion of 26.2%. This difference corresponded to a Cohen’s *h* of 0.61, suggesting a moderate‐to‐large effect size. However, the estimated statistical power was only 38.0%, well below the conventional 80% threshold.

Thus, the study may have been underpowered to detect meaningful differences, particularly in subgroup analyses involving injured participants and IES‐R outcomes. Consequently, nonsignificant findings should not be interpreted as evidence of no association but rather as inconclusive results due to limited sample size and small numbers within strata.

Finally, a post hoc power analysis was conducted to assess whether the study had sufficient statistical power to detect the observed difference in injury occurrence between dentists and nondentists, as shown in Table 10.

**TABLE 10:** Post hoc power analysis for injury occurrence by dentist status.

Parameter	Result
Observed comparison	Dentists vs. nondentists
Observed injury proportion among dentists	55.6%
Observed injury proportion among nondentists	26.2%
Effect size	Cohen’s *h* = 0.61
Post hoc statistical power	38.0%
Interpretation	Underpowered to detect meaningful differences

## 4. Discussion

The findings of this study indicate that needlestick and sharps injuries, beyond their objective biological risk, constitute potentially traumatic events capable of producing psychological sequelae among PHC professionals. The presence of symptoms related to intrusion, avoidance, and hyperarousal—together with reports of fear and anxiety—reinforces the understanding of these incidents as occupational stressors shaped by subjective, organizational, and institutional dimensions. However, the quantitative findings must be interpreted cautiously, as the study was not powered to provide definitive inferential conclusions, particularly in subgroup analyses involving injured professionals. Therefore, the results should be understood as exploratory and hypothesis‐generating rather than confirmatory.

The findings should not be interpreted as definitive evidence of causal or predictive associations. Rather, they indicate preliminary patterns suggesting that needlestick and sharps injuries in PHC may involve biological, psychological, behavioral, and organizational dimensions that deserve further investigation in larger and more robust studies.

In addition, the findings indicate that needlestick and sharps injuries remain an occupational health problem, consistent with the literature [40, 55], which describes them as a persistent public health challenge with direct consequences for worker safety and quality of care. The self‐reported occurrence of injuries in approximately one‐third of the sample (30.2%) underscores the magnitude of this issue, even in contexts where specific worker‐protection regulations exist, such as NR‐32 [56, 57]. NR‐32 establishes guidelines to protect the health and safety of workers in health services by preventing occupational risks of biological, chemical, physical, ergonomic, and accident‐related nature.

The higher proportion of injuries among dentists (71.4%) and nursing technicians (35.0%) suggests that occupational risk varies according to the nature of tasks performed, particularly those involving frequent handling of sharps and invasive procedures, although it should be noted that other categories also handle sharp devices, such as needles. This finding aligns with studies indicating greater exposure among professional groups directly involved in continuous technical practices, often carried out under time pressure, workload overload, and structural constraints [40, 58, 59]. Nevertheless, because the professional subgroups were small, these differences should not be interpreted as definitive evidence of higher risk in one category but rather as a clinically relevant pattern that requires confirmation in larger samples.

Although hepatitis B vaccination coverage was highly consistent with other Brazilian studies [60], relevant gaps were identified in anti‐HBs serologic testing and, especially, in guidance to perform this test. The low proportion of adequately informed professionals (39.6%) and reports of not knowing the need for the test or where to obtain it reflect weaknesses in institutional occupational health surveillance and shortcomings in continuing education and in the management of worker health, as discussed by Costa et al. [61] and corroborated by Rocha et al. [62]. Thus, vaccination coverage alone should not be interpreted as sufficient protection if it is not accompanied by postvaccination serological monitoring, systematic guidance, and institutional follow‐up.

Regarding risk perception, most participants reported feeling vulnerable to acquiring diseases at work, and the conditions most frequently cited as possible through cross‐infection (HIV, viral hepatitis, and tuberculosis) appropriately reflect the biological risks present in routine health service practice [60]. However, the fact that more than half did not consider splashes of body fluids to constitute an occupational accident suggests a trivialization of certain forms of exposure—a phenomenon described in other studies [59, 63] and associated with the normalization of risk in the workplace.

This finding is particularly important because it suggests that occupational risk may have become normalized within the daily routine of PHC services. When body fluid splashes are not recognized as occupational accidents, workers may be less likely to notify exposures, seek postexposure care, or demand institutional protection. In this sense, the result does not merely reflect individual lack of knowledge but may indicate gaps in the local organizational culture of safety, including insufficient continuing education, weak reinforcement of postexposure protocols, and limited institutional communication regarding what constitutes occupational exposure. Therefore, the municipality studied should consider this finding as evidence of a training and surveillance gap, rather than as an isolated perception among workers. In the local context, this finding may indicate a specific training gap regarding the definition of occupational exposure and the correct flow of postexposure care in the municipal PHC network.

The qualitative analysis deepened understanding of the psychosocial consequences associated with needlestick and sharps injuries. Participants’ statements revealed intense feelings of fear, anxiety, and insecurity, particularly during the waiting period for serologic test results—findings widely reported in the literature [20, 24, 25] as characteristic of postexposure emotional distress. This suffering tends to be intensified by the absence of institutionalized psychological support, a reality also observed in this study, in which participants reported not receiving psychological follow‐up after the accident.

Assessment of psychological impact showed that a relevant proportion of injured professionals presented IES‐R scores compatible with clinically significant post‐traumatic stress symptoms or elevated psychological distress, depending on the cutoff adopted. This clinical interpretation strengthens the relevance of the findings since the IES‐R results should not be viewed only as numerical scores but as indicators of possible psychological suffering after occupational exposure. Although mean scores indicated predominantly mild‐to‐moderate symptomatology, the frequency of symptoms suggests that psychological consequences should not be neglected.

Descriptive analysis of the subscales showed higher intensity of avoidance and intrusion symptoms, particularly among nurses and oral health assistants, indicating recurrent thoughts and avoidant behaviors related to the traumatic event. These results are consistent with Fernandes et al. [64] and supported by Parola et al. [65], who report that professionals more continuously involved in direct care may experience greater emotional burden in risk situations. However, subgroup‐specific mean scores should be interpreted with particular caution, as some strata included very small numbers of participants. Therefore, these values cannot be assumed to represent collective trends and should be considered only as descriptive indicators.

Work Psychodynamics provides important contributions to interpret these findings. Dejours, as systematized by Lancman and Sznelwar [66], argues that suffering at work emerges when there is a mismatch between organizational demands and workers’ subjective coping possibilities. Specifically, in needlestick/sharps injuries, individual blame, risk normalization, and lack of institutional recognition of the lived suffering tend to intensify this mismatch, favoring the transformation of suffering into psychological illness. From this perspective, the psychological burden identified in this study should not be interpreted exclusively as an individual reaction but also as a consequence of institutional arrangements that may fail to provide recognition, support, and structured postexposure care.

Notably, the absence of statistically significant associations between IES‐R scores and professional or demographic variables may be partly explained by the small sample size within the analyzed strata. This limitation does not mitigate the weakness of the evidence; rather, it reinforces that nonsignificant findings are inconclusive and may reflect Type II error. Therefore, the absence of statistical significance should not be interpreted as an evidence of absence of psychological impact or absence of association. Nevertheless, the observed patterns are clinically and organizationally relevant, reinforcing the need for preventive strategies and universal psychological support regardless of professional category, age, or length of service [65–70].

This study has important methodological limitations that must be explicitly considered. First, the use of a nonprobabilistic convenience sample may have introduced selection bias, limiting the external validity and generalizability of the findings to other municipalities, services, or professional populations. Second, because information on previous occupational injuries was self‐reported, recall bias may have occurred, particularly among professionals with longer time since exposure. Third, social desirability bias may have influenced responses related to biosafety practices, accident notification, vaccination status, and postexposure conduct, as participants may have reported behaviors perceived as more appropriate. Fourth, vaccination and anti‐HBs testing information were self‐reported and were not systematically verified through vaccination records or serological documentation. Finally, the small number of injured participants limited the stability of regression models and reduced the statistical power to detect meaningful differences.

For these reasons, the quantitative findings should be interpreted as preliminary and exploratory. The study contributes by identifying potential risk patterns and highlighting psychological and institutional dimensions of occupational exposure, but it does not provide definitive causal or predictive evidence. Future studies should include larger probabilistic samples, multicenter designs, documentary confirmation of vaccination and serological status, and longitudinal follow‐up to better evaluate psychological outcomes after occupational exposure.

Considering the exploratory nature of this local mixed‐methods study, future research should build upon these preliminary findings through multicenter designs involving different municipalities, health service settings, and professional categories. Studies with precalculated statistical power, larger sample sizes, and probabilistic sampling strategies are recommended to improve the precision of estimates, reduce selection bias, and strengthen the external validity of the results. Such methodological improvements would allow more robust assessment of the biological, psychological, behavioral, and organizational dimensions associated with needlestick and sharps injuries in PHC, as well as more reliable identification of factors related to postexposure psychological distress.

Finally, the findings highlight the need to strengthen institutional policies that integrate biosafety measures, continuing education, occupational health surveillance, postexposure referral, and mental healthcare. In practical terms, municipalities should reinforce permanent education on what constitutes occupational exposure, standardize postexposure protocols, ensure access to anti‐HBs testing and reference services, encourage notification without blame, and provide psychological support after accidents. Recognizing that needlestick and sharps injuries produce repercussions that go beyond immediate biological risk is essential for improving worker protection and quality of care in PHC.

## 5. Conclusion

Needlestick and sharps injuries are relevant occupational events in PHC, affecting approximately one‐third of the professionals assessed. The higher proportion of injuries observed among dentists should be interpreted cautiously due to the small subgroup size, but it may indicate greater proportional exposure among professionals who routinely perform invasive procedures and handle sharps.

Despite the high self‐reported hepatitis B vaccination coverage, gaps were identified in anti‐HBs testing and in professional guidance regarding postvaccination serological monitoring. These findings indicate that vaccination coverage alone is not sufficient to ensure occupational protection if it is not accompanied by systematic serological follow‐up, institutional guidance, and accessible postexposure protocols.

The findings suggest psychological sequelae associated with needlestick and sharps injuries, including symptoms of intrusion, avoidance, and hyperarousal, as well as reports of fear, anxiety, insecurity, and sleep disturbance. Although no statistically significant associations were found between IES‐R scores and sociodemographic or professional variables, these findings should be interpreted cautiously because of the small number of injured participants and limited statistical power.

From a practical perspective, PHC services should move beyond generic biosafety recommendations and implement structured institutional actions. These should include periodic biosafety training, clear definition of what constitutes occupational exposure, systematic reporting of needlestick, sharps, and splash‐related accidents, immediate referral to reference services, postexposure serological follow‐up, monitoring of hepatitis B vaccination and anti‐HBs status, availability of PPE and safety‐engineered devices, and nonpunitive reporting systems.

As a local exploratory study with a nonprobabilistic sample and a small number of injured participants, these findings should be interpreted with caution and should not be generalized without considering the methodological limitations. In addition, psychological support should be incorporated into postexposure care pathways since occupational accidents involving biological material may produce emotional distress and symptoms compatible with traumatic stress. In practical terms, municipal PHC services should establish clear postexposure care pathways, including immediate accident notification, referral to reference services, anti‐HBs monitoring, serological follow‐up, nonpunitive reporting, and psychological screening or referral after exposure. Therefore, prevention and management of needlestick and sharps injuries in PHC should integrate biological protection, occupational surveillance, continuing education, and mental healthcare to improve worker safety and quality of care.

## Funding

This study was funded by Fundação de Amparo à Pesquisa do Estado de São Paulo, 25/04482‐7.

## Conflicts of Interest

The authors declare no conflicts of interest.

## Data Availability

The data that support the findings of this study are available from the corresponding author upon reasonable request.
